# Is Cognitive Flexibility Equivalent to Shifting? Investigating Cognitive Flexibility in Multiple Domains

**DOI:** 10.5334/joc.403

**Published:** 2024-10-10

**Authors:** Thea Ionescu, Robert L. Goldstone, Doris Rogobete, Mihaela Taranu

**Affiliations:** 1 Psychology, Universitatea Babes-Bolyai, Cluj-Napoca, RO; 2 Psychological and Brain Sciences, Indiana University, Bloomington, US; 3 Department of Digital Design and Information Studies, Aarhus University, Aarhus, DK

**Keywords:** cognitive flexibility, set-shifting, domain-specificity

## Abstract

In the present exploratory study we investigate whether cognitive flexibility is a unitary mechanism underlying flexible behaviours across many domains or a domain-specific capacity. The literature on cognitive flexibility is divided into several research lines that do not converge. The most prominent one considers flexibility an executive function that represents the ability to switch among rules or tasks. In other research traditions it is associated with distinct components, such as the capacity to place an item into many categories (in creativity tests) or a characteristic of different cognitive or perceptual processes (e.g., flexible language use, flexibility in mathematics, perceptual flexibility). To determine whether flexibility in different domains relies on a general shared mechanism, 221 subjects from two countries (The United States and Romania, mean age 19.52 years) were tested online with several measurements from four different domains of investigation: language, mathematics, perception, and executive functions (specifically, set shifting). All tasks required some form of cognitive flexibility. In addition, we measured math anxiety to see how this relates to mathematical flexibility. The results show very few and small significant partial correlations among the tasks. They also highlight that there is no unitary overarching “executive” factor. The most prominent common factor was speed of processing for mathematical and language response times. Shifting does not seem to be a mechanism that underlies flexibility in all the investigated domains. While we acknowledge the need for replication of this study, the data suggest that the construct of shifting does not exhaust the notion of flexibility as it arises across cognitive domains.

While highly praised as an essential human characteristic (Deák, 2003; Kraft et al., 2020) cognitive flexibility still lacks a straightforward conceptualization in the literature (Ionescu, 2012, 2017). It has several non-convergent definitions, and it has been studied via multiple research paradigms that assume different constructs and involve different cognitive processes. For example, in one research tradition, cognitive flexibility is construed in terms of set-shifting, the ability to switch rapidly between different rules, tasks or mental sets (Diamond, 2006; Uddin, 2021; Ueltzhöffer et al., 2015). In another, it is equated with the broadness of knowledge, as measured, for example, by the number of categories that one can use to classify an object (Dietrich & Kanso, 2010). In yet another, it is a cultural dimension that expresses the willingness to adapt to others and learn from them (Minkov & Kaasa, 2021). The questions that immediately come to mind after perusing the literature are: which one, if any, of these characterizations captures “true” flexibility? How can we understand the mechanisms underlying cognitive flexibility and foster its development when there is no agreement about what it is?

The present endeavour is an exploratory study of flexibility in different domains of investigation with the aim to uncover the commonalities and differences when one’s behaviour is flexible in those domains. We use the term “domains” to refer to broad areas of investigation in psychology, and not to specific domains of knowledge. Specifically, we will investigate rule-use flexibility, language flexibility, perceptual flexibility, and mathematical flexibility, exploring the possibility that flexibility is a property of the cognitive system and not one particular ability or mechanism (Ionescu, 2012). To the extent that performances across these tasks are associated, a common cognitive mechanism of flexibility will be implicated. To the extent that they are dissociated, separate processes underlying flexibility in particular domains are indicated. The paper is organized as follows: first, we briefly present some of the existing research traditions on cognitive flexibility; second, we describe a study that looks for associations among flexible responses in different domains; in the end, we discuss the findings and the nature of cognitive flexibility.

## Cognitive flexibility: current conceptualizations

Different research traditions highlight quite distinct aspects of cognitive flexibility. The most frequently encountered proxy for cognitive flexibility is set-shifting. In the literature on executive functions (EF), set-shifting represents the ability to switch between rules or task sets (Best et al., 2009; Garon et al., 2008; Huizinga et al., 2006; Miyake et al., 2000; Monsell, 2003) and it is one of the three most studied EFs, namely inhibition, working memory, and shifting (Diamond, 2006, 2013). Set-shifting has been found to be predictive of academic success and adjustment in the face of life adversities, and as such it is considered an essential cognitive mechanism for optimal cognitive functioning across the life span (Müller & Kerns, 2015; Uddin, 2021). While not all researchers equate this set-shifting ability with cognitive flexibility (see for example the research on cognitive control, Kriete et al., 2013; Politakis et al., 2022; Rougier et al., 2005), many treat the two interchangeably (Cragg & Chevalier, 2012; Diamond, 2006, 2013; Uddin, 2021). What is most concerning is that in some studies there is no definition provided for flexibility; the main focus is set-shifting defined as above, and only in discussions do authors refer to cognitive flexibility as another name for set-shifting. In other words, after analyzing set-shifting performance, the authors draw conclusions about cognitive flexibility more broadly, tacitly assuming that they are equivalent. In a recent review, Howlett and her colleagues (2022) show that task-based performance in set-shifting and self-reports about set-shifting do not correlate. As such, they suggest more caution when speaking about the same underlying mechanism and further studies to disentangle what constitutes cognitive flexibility.

A second line of studies that refers to flexibility is from research in creativity, which considers flexibility as a measure for divergent thinking (together with fluency, originality and elaboration, Guilford, 1967). In the well-known Alternative Uses Test (AUT, Guilford, 1978) flexibility is operationalized as the number of categories from which one draws different uses for a given object. For example, if one is asked to produce as many uses as possible for a brick and the participant produces five ideas, she will be given a high score of flexibility when the ideas are as diverse as possible. To our knowledge, there aren’t many attempts to define flexibility per se in this research tradition. It is also the case that the term here is “flexibility” and not “cognitive flexibility”. Flexibility within the divergent thinking literature does not have unique trait variance beyond fluency and originality, since flexibility is predicted by fluency and originality and is dependent on working memory and mental speed (Weiss & Wilhelm, 2022). Similar to these authors, we agree that improvements in the measurement of flexibility are needed to fully understand what flexibility means within the creativity research tradition.

A third line of studies involves the investigation of representations, of various cognitive processes, and of perceptual processes and their flexibility (Flavell et al., 1983; Mareschal & Quinn, 2001; Nguyen & Murphy, 2003; Sapp et al., 2000; Sloutsky & Fisher, 2008). For instance, studies on categorization describe flexible categorization when one exemplar is assigned to different appropriate categories (Ross & Murphy, 1999). Several studies show that children can categorize flexibly if the context affords it (e.g., depending on instructions, Deák & Bauer, 1996; Ionescu, 2019; Waxman & Namy, 1997) and that adults’ expertise influences the use of different types of information (Shafto & Coley, 2003). Similarly, studies on language investigate its flexible use in various contexts (Plunkett, 2006). For example, children begin very early to use a known word in novel settings, even before their third year of life (Naigles et al., 2009). Flexibility in mathematics has been operationalized in terms of the ability of a reasoner to develop, compare, and contrast multiple ways of solving the same problem (Rittle-Johnson & Star, 2009; Star & Seifert, 2006). Prior to solving a math problem, flexibility is also involved in reorganizing a mathematical object to emphasize different structures, such as sub-expressions in an algebraic equation or two-dimensional shapes in a complex diagram (Yu, Landy, & Goldstone, 2018). Flexibility also manifests in perception when, for example, during the observation of ambiguous figures our perceptual interpretation spontaneously changes even though the visual information stays unchanged. Classical examples of ambiguous figures are images such as the Necker Cube, Rubin’s face/vase, and Boring’s young girl/old woman, which are investigated in perceptual psychology, neuroscience, and computational modeling studies. The perceptual alterations happening when observing such images is called bistability. All in all, in this third group of research, flexibility is considered to be a characteristic of the investigated process, without a focus on flexibility or cognitive flexibility as a broader concept to be defined.

Yet another line takes a much broader perspective. In this research group, one can find flexibility linked to openness to experience in personality studies (Chung et al., 2012), or to accepting one’s thoughts in the Acceptance and Commitment Therapy (Scott et al., 2016), or even as a new cultural dimension of Hofstede’s model, flexibility (vs monumentalism, Minkov & Kaasa, 2021). These too are not referred to as “cognitive flexibility” but rather as “psychological flexibility”. However, by generally using only the word “flexibility”, one can be tempted to question whether there is a genuine difference between “cognitive flexibility” and “psychological flexibility”?

All of the four above mentioned research lines pertain, one way or another, to flexibility but, as we can easily see, they do not converge. Are all of them appropriately interpretable as “cognitive flexibility”? Do they refer to the same mechanism responsible for flexible answers? Is there such a single mechanism that underlies these different conceptions? These are the general questions that guide our exploratory study. A recent integrative account aims to reach a common view of flexibility and considers it to be a property of the cognitive system or its subsystems (Ionescu, 2012, 2017). One mechanism seems not to be enough for flexible behaviors to appear (O’Reilly, 2006) and flexible behaviors are also heavily influenced by context (Miller & Fusi, 2013; Perry, 2015; Stokes et al., 2013; Thomas et al., 2012). As such, cognitive flexibility might arise when several internal mechanisms interact well, like shifting or attentional mechanisms to name a few, and when these are also well adjusted to contextual cues, like the demands of a new rule or problem (see Figure 1 in Ionescu, 2012). Importantly, one prediction that follows is that shifting alone will not suffice for flexibility to emerge. While shifting is essential for flexibility, because one must switch perspectives for changing behavior in order to adapt or find a novel approach, one also needs other mechanisms, like inhibition, goal maintenance, or error monitoring, together with a robust knowledge base from which to activate distinct perspectives. One comprehensive review of the development of executive functions agrees that because flexibility relies on a variety of processes it may be better conceptualized as an emergent property (Müller & Kerns, 2015). We adopt this approach here, namely the idea that cognitive flexibility might be a property of the cognitive system (Ionescu 2012, 2017), and explore whether performance in various domains rely on different configurations of mechanisms in those particular areas (what we will call “domain-specific”) or rather on a similar mechanism (like set-shifting as a core “domain-general” cognitive mechanism).

**Figure 1:**
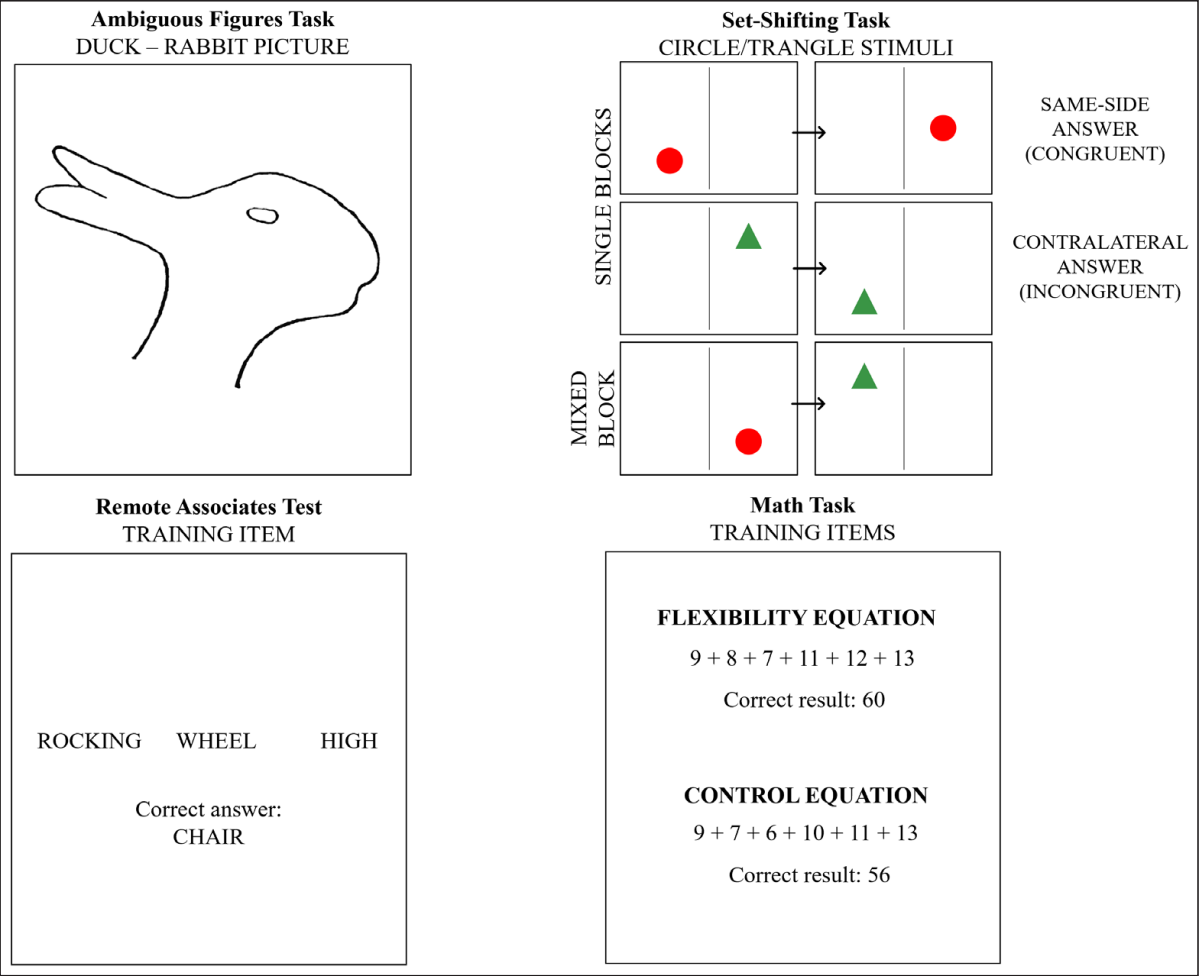
Item examples from each of the four tasks.

## The present study

The aim of the present study is to investigate whether there is a common underlying mechanism responsible for flexibility across domains or, instead, flexibility is a domain-specific characteristic. To this end, we analyzed flexibility with multiple tasks in different domains.

We have chosen four domains – executive functions, language, perception, and mathematics – and devised or adapted four tasks that require flexibility, i.e. identifying a new perspective and using it for solving the task. To our knowledge, no previous study has combined tasks in all these areas to investigate flexibility, therefore it is an exploratory study. We assessed shifting as an executive function with an adapted Dots – Triangles task (Huizinga et al., 2006) that specifically assesses rule-use flexibility or rule-switching, i.e., changing one’s response according to different rules. Even though most adult shifting tasks use letters or numbers as stimuli, we used geometrical shapes and their position on the screen as main indicators of the rules to avoid potentially inflated correlations to the mathematical and language tasks in our experiment due to the similarity of stimuli.

For language flexibility, we have chosen to use a sample of items from the Remote Associates Test (RAT; Mednick & Mednick, 1967), a widely used creativity test that resembles insight problem solving (Cai et al., 2009; Cunningham et al., 2009). Because this test is based on finding associations among words (i.e., find a fourth word that relates to the other three given words) we used it as reflecting flexibility with words (i.e., coming up with new words associated with an initial set of words).

For perceptual flexibility, we used several ambiguous figures (images with multiple interpretations) to assess how people’s perception switches among different interpretations across different types of figures. Bistable images have been used to study insight and creativity, as people who are able to discover the different interpretations residing within an ambiguous image also tend to be good at creative problem solving (Laukkonen & Tangen, 2017).

For flexibility in mathematics, we employed a task requiring participants to solve relatively simple algebraic problems. In one condition, participants must solve them by “brute force” calculation, for example “5 + 17 + 2 + 11 + 12 + 4 = ?”. In a second condition, they can take advantage of shortcuts to dramatically reduce the calculations needed (Benjamin & Shermer, 2006), for example realizing that “5 + 6 + 7 + 13 + 14 + 15 = ?” can be rearranged as “(5 + 15) + (6 + 14) + (7 + 13)” and so is 20 * 3 = 60. The ability to flexibly reorganize mathematical expressions is a core competency for mathematical proficiency and is positively associated with both high stakes tests of mathematical reasoning and being able to generate and use mathematical models for reasoning in science and engineering (Kilpatrick et al., 2001; Schoenfeld, 1992). We also gave participants an instrument to assess their anxiety about mathematics (Hopko et al., 2003) to explore the possibility that when people feel uncertain, anxious, or negative when asked to solve mathematics problems then they may show a reduced tendency to flexibly explore the possibility of reorganizing a mathematical expression to reveal a shortcut (Ramirez et al., 2018).

Based on the distinct literatures presented above we expect that: (a) If there is a high correlation among all tasks, a common mechanism can be assumed for all types of flexible responses (possibly set-shifting); (b) If there is no correlation among the tasks, flexibility can be assumed to be a domain-specific capacity.

## Method

### Design

A mixed design was employed, with within-task manipulations as described below, and correlations across tasks.

### Participants

223 young adults were initially tested with all the tasks, 94 from the United States (USA sample) and 129 from Romania (RO sample). They were first year undergraduate students at two universities, one in the USA and one in Romania. Given the exploratory nature of the present study and the fact that the investigated effects are not found as such in the previous literature, we did not conduct a power analysis but estimated the sample size based on the feasibility of our study.

Two subjects (one in the USA sample, one in the RO sample) were eliminated due to erratic responses and not respecting the instructions in all tasks. The remaining 221 subjects were included in the analyses (M age = 19.52 years, SD = 2.67; 47 male subjects, 165 female subjects, 2 “other” gender subjects, 7 not reporting their gender – see Table 1).

**Table 1:** Descriptive Statistics For the Overall Sample and by Country.


TASK SCORE	COUNTRY = ROMANIA (N = 128)					COUNTRY = USA (N = 93)					OVERALL SAMPLE (N = 221)				
		
	*N*	*M*	*SD*	*MIN*	*MAX*	*N*	*M*	*SD*	*MIN*	*MAX*	*N*	*M*	*SD*	*MIN*	*MAX*

Sex															

Male	10					37					47				

Female	115					50					165				

Other	1					1					2				

Age (years)	125	19.82	3.34	18.00	40.00	88	19.09	1.06	17.00	24.00	213	19.52	2.67	17.00	40.00

SH-switching cost	126	0.08	0.10	–0.13	0.66	88	0.09	0.07	–0.05	0.44	214	0.09	0.09	–0.13	0.66

SH-mixing cost congruent	125	0.22	0.11	–0.11	0.65	89	0.18	0.13	–0.32	0.78	214	0.20	0.12	–0.32	0.78

SH-mixing cost incongruent	124	0.15	0.10	–0.15	0.63	89	0.11	0.10	–0.38	0.34	213	0.13	0.10	–0.38	0.63

RAT-accuracy	127	6.80	3.11	1.00	15.00	87	8.52	3.25	1.00	15.00	214	7.50	3.27	1.00	15.00

RAT-RT	127	15.10	8.17	3.15	45.59	87	11.70	9.14	4.29	74.32	214	13.72	8.72	3.15	74.32

AF-sum of switches	128	5.06	1.00	2.00	6.00	92	4.62	1.24	1.00	6.00	220	4.88	1.13	1.00	6.00

AF-switch rate	128	18.63	28.15	0.00	182.50	92	16.58	20.27	0.00	135.00	220	17.77	25.13	0.00	182.50

AF-switch rate RT	128	1.35	1.73	0.10	10.05	92	1.57	2.92	0.12	21.38	220	1.44	2.30	0.10	21.38

MATH-overall accuracy	128	13.36	1.78	9.00	16.00	84	12.68	2.90	2.00	16.00	212	13.09	2.31	2.00	16.00

MATH-flexibility accuracy	128	7.01	0.98	4.00	8.00	84	6.55	1.42	2.00	8.00	212	6.83	1.19	2.00	8.00

MATH-control accuracy	128	6.35	1.23	3.00	8.00	84	6.13	1.78	0.00	8.00	212	6.26	1.47	0.00	8.00

MATH-accuracy difference	128	0.66	1.32	–3.00	4.00	85	0.42	1.39	–3.00	4.00	212	0.56	1.35	–3.00	4.00

MATH-overall RT	128	16.52	5.48	6.60	34.14	84	14.41	5.12	4.36	29.80	212	15.68	5.43	4.36	34.14

MATH-flexibility RT	128	15.31	5.56	6.13	32.35	84	13.83	5.50	3.85	28.03	212	14.73	5.57	3.85	32.35

MATH-control RT	128	18.34	5.95	7.45	36.94	83	15.59	5.33	5.83	29.80	211	17.26	5.86	5.83	36.95

MATH-RT difference	128	3.05	3.26	–6.91	12.74	84	1.97	3.34	–5.10	11.49	212	2.62	3.33	–6.91	12.74

AMAS avg. score	128	2.89	0.70	1.56	4.89	92	2.99	0.92	1.00	4.89	220	2.93	0.80	1.00	4.89


*Note*. AF = Ambiguous Figures Task; RAT = Remote Associates Test; SH = Set-Shifting Task; MATH = Math Task; RT = response time; AMAS = Abbreviated Math Anxiety Scale.

In the Romanian sample, 96.9% reported Romanian as their native language and 98.4% reported a Psychology major. A few participants did not report native language (3.1%) or major (1.6%). The USA sample was more diverse. The majority reported English as their native language (84.9%%), followed by Spanish (3.2%), Tamil, Chinese and Croatian (1.1% each). A few participants did not report native language (8.6%). Regarding major, most participants reported Psychology (13.98%), Business and finance (15.05%) or Biology (12.90%), followed by smaller percentages of Science (9.68%), Exercise and nutrition (7.53%), Nursing (7.53%), Media and Marketing (6.45%), Public health (5.38%), Arts and dance, Criminal justice and Informatics and Design (3.23% each), International studies and social work (1.1% each). A few participants were undecided regarding major (2.15%) or did not report major (7.5%).

Ethics approval was obtained at both sites. Each participant has given consent to participate and have received course credit for participation.

### Tasks

#### Technical aspects

All tasks were constructed using PsychoPy 2022.1.1, version: 3.8.10, and were hosted and run online, using Pavlovia.org (https://pavlovia.org/). A single experiment was constructed to encompass all four tasks, along with the informed consent, demographics information and the Abbreviated Math Anxiety Scale (AMAS) (see below).

#### Linguistic aspects

The experiment ran in English for the USA sample and in Romanian for the Ro sample. Instructions were carefully translated and piloted in order to make sure that the same meaning was conveyed for each task in both languages. For the RAT, nationally validated and normed versions were used for each country (Bowden & Jung-Beeman, 2003; Olteţeanu et al., 2019). The AMAS items were also translated into Romanian and piloted with a different small sample for item comprehensiveness and face validity.

##### Set-Shifting – the adapted Dots Triangles Task (SH Task)

A modified version of the Dots-Triangles task was constructed using the stimuli described in Crone et al. (2006) and the task structure used by Huizinga et al. (2006) (see Figure 1 for an example). Stimuli consisted of one red circle or one green triangle approximately 4 cm in size each, presented on a white background, either on the left or the right side of a vertical 6 cm long black line, position that was relevant for the response. On each side, the shape could appear in the upper corner, lower corner or in the center, which was irrelevant for the response. Participants had to respond when one of the two shapes appeared on screen according to the following rules: for circles, pressing the button on the same side with the position of the circle on the screen (left or right part of the screen; congruent answer); for triangles, pressing the button on the contralateral position of the shape (left for right screen triangles and right for left screen triangles; incongruent answer). Participants responded by pressing the P (right) or Q (left) keys as fast as they could. All stimuli were presented for a maximum of 3500 ms or until the participant responded, with an interstimulus interval randomly selected between 500 ms and 600 ms. The task had 3 parts, completed in the same order by all participants: two single-task parts (exclusively circle trials, followed by exclusively triangle trials), and one mixed-task part (a mix of circles and triangles trials). Each of the single-task parts had a training block of 30 trials and a test block of 54 trials. The mixed block had 48 training trials and 96 test trials. Reaction times (RTs; measured in seconds) were trimmed following the procedures used in the literature: those faster than 120 ms or longer than the individual mean plus 2.5 SDs were eliminated. Mean reaction times were calculated with the remaining data for each of the following types of trials in order to calculate the final scores: single-task congruent (circles) and incongruent (triangle), mixed task congruent and incongruent and mixed task repeat (two successive trials with the same response rule – e.g., two circles trials) and switch trials (two successive trials where a change of the response rule occurs – e.g., a triangle trial followed by a circle one). Final participant scores for this task were: (1) Switching cost (SH – switching cost) and (2) Mixing costs for congruent and incongruent stimuli (SH – mixing cost congruent, SH – mixing cost incongruent). The original tasks on which this test was based have shown adequate construct validity as indicated by relevant relationships and factor convergence with tasks that measure similar constructs, such as the Number-pinyin task (Xu et al., 2013), the Local-Global and the Smiling Faces Tasks (Huizinga et al., 2006), which measure set-shifting ability with various types of stimuli. Switching costs and mixing costs also show moderate reliability when measured in different types of tasks (Segal. et al., 2021).

##### Language Flexibility – the Remote Associates Test (RAT)

Language flexibility was measured using a sample of triads from the Remote Associates Test (RAT) (see Figure 1 for an example and the Appendix for the complete list of stimuli). In this task, participants were presented with a total of 16 triads of words, one by one – one training triad and 15 test triads. For each of them, participants had to find a fourth word that made meaningful compound words or phrases with each of the three words. For example, for the triad {cottage, swiss, cake} the correct answer would be ‘cheese’ because ‘cottage cheese’, ‘swiss cheese’, and ‘cheesecake’ are all common two-word phrases. Each triad was presented on the screen until the participant reached a solution and responded by clicking a button and typing in the solution word. Participants received feedback as to whether their response was correct or incorrect after each answer. Triads were chosen to lie between 25% and 75% performance levels in the studies assessing the norms for these countries (Bowden & Jung-Beeman, 2003; Olteţeanu et al., 2019). Both the Romanian and the English versions of the task showed adequate internal consistency (Cronbach’s alpha US = 0.72; Cronbach’s alpha RO = 0.72). The relevant scores for this task were: (1) Accuracy (RAT – accuracy; the number of correct responses; maximum 15 points) and (2) Response time (RAT – RT; the median interval between the moment when the triad appeared on the screen and the moment when the participant submitted his answer; measured in seconds).

##### Perceptual Flexibility – Ambiguous Figures Task (AF Task)

The Ambiguous Figures Task included seven ambiguous figures, one for training (Duck/Rabbit image) and six for the testing phase with two images from each of the following main types of ambiguous figures: meaning content reversals (a. Eskimo/Indian, b. Squirrel/Swan); figure ground reversals (c. Monkey in a tree/Tiger, d. Saxophone player/Woman’s face); and perspective reversals (e. Necker’s cube, f. Schröder reversible staircase; see Figure 1 for an example and the Appendix for the complete list of images). The images originate from the collection of Snodgrass & Vanderwart (1980). All images were scaled to the same dimension (1280 × 1024 pixels) and were presented to the participants as static black-and-white figures. As far as we know, validity has never been calculated for ambiguous figure images. During ambiguous image interpretation tasks, perceptual fluctuation rates vary considerably between individuals (Kleinschmidt et al, 2012) and even across different visual stimuli since the same individual can have different switching rates for the different stimuli (Cao et al, 2018). Despite the high individual differences manifested across visual stimuli, participants respond consistently within stimuli and even across visual or auditory modalities (Denham et al, 2018), indicating that perceptual switching is a consistent predictor of individual differences in perception.

For each figure, the image was first presented on the screen for 10 seconds and participants had to write what they saw as soon as they identified one interpretation. Next, the same image was presented again for up to 50 seconds and participants were asked to type a second interpretation as soon as they saw it. If participants were not able to identify both interpretations after the first two presentations of the image, they were given the two interpretations. After this, the picture was presented a third time, for 60 seconds, and participants had to report continuously during this interval whenever they switched between the two interpretations, using two buttons on the keyboard (P and Q). All participants first completed the training stimulus, followed by the six test-stimuli, in randomized order. Three types of scores were calculated: (1) Sum of switches or the ability to change interpretations across all 6 images (AF – sum of switches; total number of images for which the participant identified two correct interpretations in the first two parts of the testing phase; maximum 6 points); (2) Switching rate (AF – switch rate; the median number of times participants switched between the two interpretations of an image during the subsequent 60 seconds); (3) Reaction time for the switching rates (AF – switch rate RT; median reaction times for the switching rate; measured in seconds).

##### Math Flexibility – the Math Task

The Math Task consisted of a total of 18 equations that were presented on the screen, one by one. They were built based on the stimuli used in Yu, Landy, and Goldstone (2018) (see Figure 1 for an example and the Appendix for the complete list of stimuli). Of these, half were “flexibility equations”, which allowed a faster resolution if the participant used grouping or other shortcut strategies (e.g., grouping terms that resulted in 20s and adding the 20s in the end; 9 + 8 + 7 + 11 + 12 + 13), and the other half were “control equations”, that allowed sequential resolution, using basic mathematical principles (e.g., PEMDAS; 9 + 7 + 6 + 10 + 11 + 13). Participants first completed two training equations (one flexibility and one control), followed by 16 test equations, presented in randomized order for each participant (8 flexibility and 8 control). An equation was presented on the screen for a maximum of 60 seconds or until the participant reached a solution, clicked a button, and typed in their answer. After submitting the response, they received feedback on their performance, both in the training and in the test phase. At the end of the task, participants were required to type in the strategies they used during the task. Since it was a task constructed de novo for the purpose of this study, we calculated the internal consistency as a measure of achieved reliability. Results indicate moderate reliability for the overall accuracy score (Cronbach’s alpha = 0.61). Multiple types of scores were calculated: (1) Overall accuracy (MATH – overall accuracy; number of correctly answered equations; maximum 16 points); (2) Separate Flexibility and Control accuracy (MATH – flexibility accuracy, MATH – control accuracy; maximum 8 points for each); (3) Accuracy difference (MATH – accuracy difference; the difference in accuracy between Flexibility equations and Control equations) (4) Overall response time (MATH – overall RT; the median interval between the moment when the equation appeared on the screen and the participant submitting their answer, for correctly answered equations; measured in seconds); (5) Separate Flexibility and Control response times (MATH – flexibility RT, MATH – control RT; medians; measured in seconds); (6) Response time difference (MATH – RT difference; median difference between median response times on Control and Flexibility equations; measured in seconds).

##### Mathematics Anxiety – The Abbreviated Math Anxiety Scale (AMAS) (Hopko et al., 2003)

The AMAS consists of nine items tapping into anxiety associated with various situations that involve learning math and participating in math-related evaluations (Hopko et al., 2003). Participants report on a 5-point Likert scale (1 = low anxiety, 5 = high anxiety) the amount of anxiety they experience in each of the nine situations described by the items (e.g., “Watching the teacher work out a math problem on the board.”). Individual item responses were averaged to generate a total Math anxiety score (AMAS avg score; maximum score is 5). In the present study, the AMAS showed adequate overall reliability (alpha Cronbach = 0.86), as well as by country (alpha Cronbach US = 0.91, alpha Cronbach RO = 0.80).

## Procedure

Participants were first year undergraduate students who were informed that they can participate in this study for course credit and received the link to the experiment. Upon accessing the link, subjects were first presented with the informed consent and indicated their agreement to take part by clicking an “I agree to take part in this study” button shown on the screen. After this, they provided basic demographic information (name, age, gender, native language, major), followed by the four tasks, which unfolded one by one in random order. Lastly, they completed the nine AMAS items. Completing all steps took approximately 60 minutes.

In between the tasks, subjects could take short breaks and then click anywhere on the screen when they were ready to continue. Subjects completed all tasks online, in a single session, at a time and a place of their choice, on their personal computer. For the math and language tasks they were asked to respond as accurately and quickly as they could without using calculators, paper and pencil or other resources. For the perceptual and shifting tasks they were asked to focus their gaze on the center of the screen and move it as little as they could while answering as quickly as possible.

## Data analyses

To account for the potential effects of country and gender we performed a MANOVA test on the variables from the four tasks, which yielded a significant main effect of country (see results below). Given the result of the MANOVA, we examined whether the scores between the four tasks relate to each other while controlling for the effects of the country by using partial Pearson correlations. To this end, *country* was coded as a dummy variable (1 = USA sample, 2 = Romanian sample) and entered as a control variable in the partial correlation analysis. Partial correlations allow for the strict control of country effects during data analysis since it holds constant its effects (i.e. controls for the effect of country) on the relationship between the various variables in the study. To account for multiple hypothesis testing for the 136 partial Pearson correlations and control the false discovery rate, we subsequently adjusted the *p* values using the Benjamini-Hochberg correction. The adjusted p-values were calculated using the *p.adjust(method = “BH”)* function in RStudio (RStudio Team, 2023).

An Exploratory Factor Analysis using the Maximum Likelihood extraction method and Direct Oblimin rotation was further conducted to examine the underlying structure of the variables among the four tasks (Fabrigar et al., 1999). To examine the relationship between the AMAS and scores on the Math Task, we additionally conducted a set of partial correlations, controlling for country.

All analyses were conducted using IBM SPSS (version 26), with the exception of the p-value correction for partial correlations. Since medians protect better against the influence of skewness and outliers, we have used median response times instead of mean response times (Wilcox & Rousselet, 2018). One exception is the shifting task, where we used means and we trimmed reaction times as explained in the Instruments section, in accordance with the literature. For all but the shifting task we calculated both accuracy scores and reaction time scores in order to account for a potential speed-accuracy trade-off.

## Results

### Outliers

Data was first analyzed for overall task accuracy and the data of outlying participants was eliminated task by task manually. For the SH Task, we eliminated the data from 7 participants for the switching cost and 7 participants for the mixing costs because their task accuracy was below 50%. For the AF task, we eliminated one participant who did not complete the task. We also eliminated data from 7 participants in the RAT and 9 participants from the Math Task because all their responses were incorrect and erratic, indicating task disengagement and non-compliance to instructions. One participant failed to complete the AMAS. No imputation for replacing missing data was conducted. Rather, participants who did not have data for a certain task were not included in the analyses that were relevant for that task (See Table 1 for the exact number of participants per task score).

### Descriptive statistics

A summary of descriptive statistics for all the variables across the four tasks, separately for the two sub-samples and for the whole sample as well, can be found in Table 1.

### Country and gender differences

The multivariate results of the MANOVA showed a significant overall effect of participant country [Pillai’s Trace = .197, *F*(15, 178) = 2.92, *p* < .001, *η2* = .197], no overall effect of gender [Pillai’s Trace = .124, *F*(15, 178) = 1.68, *p* = .058, *η2* = .124] and no Gender × Country interaction [Pillai’s Trace = .094, *F*(15, 178) = 1.23, *p* = .254, *η2* = .094]. There were between subject country differences for the following variables: AF sum of switches [*F*(1, 192) = 6.18, *p* = . 014, *η2* = .031], RAT accuracy [*F*(1, 192) = 7.25, *p* = . 008, *η2* = .036], RAT RT [*F*(1, 192) = 6.41, *p* = . 012, *η2* = .032], Math overall accuracy [*F*(1, 192) = 4.68, *p* = .032, *η2* = .024] and Math flexibility accuracy [*F*(1, 192) = 8.35, *p* = .004, *η2* = .042]. Specifically, the AF sum of switches was higher for the RO sample (*M* = 5.1, *SD* = 1.0) than for the USA sample (*M* = 4.7, *SD* = 1.2). RAT accuracy was higher for the USA sample (*M* = 8.7, *SD* = 3.1) than the RO sample (*M* = 6.8, *SD* = 3.2). RAT RT was lower for the USA sample (*M* = 11.7, *SD* = 9.5) than the RO sample (*M* = 15.0, *SD* = 7.9). Math accuracy was higher for the RO sample (*M* = 13.4, *SD* = 1.7) than for the USA sample (*M* = 12.8, *SD* = 2.7). Math flexibility accuracy was also higher for the RO sample (*M* = 7.0, *SD* = 1.0) than the USA sample (*M* = 6.6, *SD* = 1.4). Country did not have a significant effect on any other variables (see complete results in the Supplementary File 1 – Table 5 – https://figshare.com/articles/figure/Supplem_1/27022282).

### Correlations

For correlations within tasks please see Table 2.

**Table 2:** Partial Correlations Between the Scores on the Four Experimental Tasks, controlled for Participant Country.


VARIABLE	SH-SWITCHING COST	SH-MIXING COST CONGRUENT	SH-MIXING COST INCONGRUENT	RAT-ACCURACY	RAT-RT	AF-SUM OF SWITCHES	AF-SWITCH RATE	AF-SWITCH RATE RT	MATH-OVERALL ACCURACY	MATH-FLEXIBILITY ACCURACY	MATH-CONTROL ACCURACY	MATH-ACCURACY DIFFERENCE	MATH-OVERALL RT	MATH-FLEXIBILITY RT	MATH-CONTROL RT	MATH-RT DIFFERENCE

**SH-switching cost**	1.000															

**SH-mixing cost congruent**	0.274***	1.000														

**SH-mixing cost incongruent**	0.255***	0.446***	1.000													

**RAT-accuracy**	–0.107	–0.050	–0.136	1.000												

**RAT-RT**	0.122	0.104	0.100	0.061	1.000											

**AF-sum of switches**	–0.011	–0.089	–0.017	0.102	–0.089	1.000										

**AF-switch rate**	–0.088	0.003	0.020	–0.026	0.040	0.042	1.000									

**AF-switch rate RT**	–0.095	0.008	0.026	0.216*	0.026	0.007	–0.207*	1.000								

**MATH-overall accuracy**	–0.117	–0.041	0.068	0.219*	0.123	0.124	–0.047	0.143	1.000							

**MATH-flexibility accuracy**	–0.132	–0.129	0.050	0.155	0.130	0.132	–0.020	0.189*	0.830***	1.000						

**MATH-control accuracy**	–0.076	0.040	0.066	0.214*	0.085	0.088	–0.058	0.073	0.896***	0.495***	1.000					

**MATH-accuracy difference**	–0.034	–0.154	–0.026	–0.093	0.019	0.019	0.046	0.084	–0.254***	0.329***	–0.658***	1.000				

**MATH-overall RT**	0.044	0.108	0.040	0.043	0.273***	–0.045	–0.048	–0.091	–0.158	–0.044	–0.212*	0.192*	1.000			

**MATH-flexibility RT**	0.020	0.093	0.037	0.009	0.236**	–0.051	–0.019	–0.075	–0.184*	–0.078	–0.224**	0.175*	0.915***	1.000		

**MATH-control RT**	0.024	0.091	0.054	0.058	0.229**	–0.047	–0.078	–0.097	–0.156	–0.028	–0.217*	0.202*	0.920***	0.771***	1.000	

**MATH-RT difference**	–0.028	–0.089	0.007	0.076	0.098	0.018	–0.168	0.009	0.035	0.016	0.043	–0.033	0.087	–0.134	0.300***	1.000


*Note*. All correlations control for Participant Country; AF = Ambiguous Figures Task; RAT = Remote Associates Test; SH = Set-Shifting Task; MATH = Math Task; Corrected p values: * p < .05, ** p < .01, *** p < .001; N = 221; for individual number of statistical units, please see df for each correlation in Table 6 Supplementary File 1.

#### Correlations between tasks

Correlations were calculated between the three variables from the SH Task (switching cost, mixing cost congruent, and mixing cost incongruent), the two variables of RAT (accuracy and RT), the three variables of the AF Task (sum of switches, switch rate, and switch rate RT), and the 8 variables of the Math Task (overall accuracy, flexibility accuracy, control accuracy, accuracy difference, overall RT, flexibility RT, control RT, and RT difference) (see Table 2).

After the p values correction, RAT accuracy correlated weakly and positively with the AF switch rate RT (*r* = .22, *p* = .011). RAT accuracy also correlated weakly and positively with the Math overall accuracy (*r* = .22, *p* = .011) and Math control accuracy (*r* = .21, *p* = .011).

The RT for the RAT Task correlated weakly and positively with the RT variables in the Math Task: overall RT (*r* = .27, *p* < .001), RT flexibility (*r* = . 24, *p* = .008), RT control (*r* = .23, *p* = .008).

The Math accuracy flexibility correlated weakly and positively with AF switch rate RT (*r* = .19, *p* = .029).

No other significant correlations were found (please find all the p values before and after correction in the Supplementary File 1, Table 6).

### Common factors among tasks

**Figure 2:**
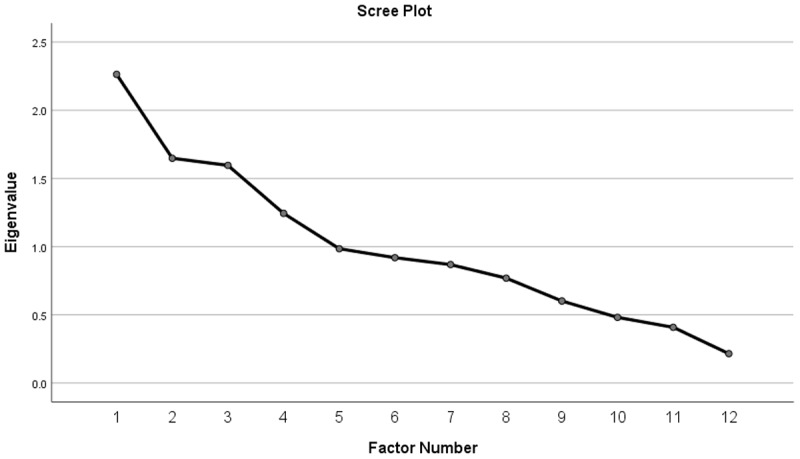
Screeplot for the entire sample (N = 200).

Four factors emerged in the Exploratory Factor Analysis with eigenvalues larger that 1 (see Tables 3 and 4): Factor 1 (eigenvalue = 2.263, 18.86% variance explained), Factor 2 (eigenvalue = 1.648, 13.73% variance explained), Factor 3 (eigenvalue = 1.596, 13.30% variance explained), Factor 4 (eigenvalue = 1.244, 10.37% variance explained). The model has an adequate goodness-of-fit (chi-square (24) = 25.085, *p* = .40). Upon inspecting the scree-plot (see Figure 2) and the goodness-of-fit indicator, we decided to retain all four emerging factors as the factorial solution.

**Table 3:** Total Variance Explained by the 4 Factors Extracted Using the Exploratory Factor Analysis.


FACTOR	INITIAL EIGENVALUES			EXTRACTION SUMS OF SQUARED LOADINGS			ROTATION SUMS OF SQUARED LOADINGS
		
	TOTAL	% OF VARIANCE	CUMULATIVE %	TOTAL	% OF VARIANCE	CUMULATIVE %	TOTAL

1	2.263	18.858	18.858	1.164	9.699	9.699	1.115

2	1.648	13.733	32.591	1.757	14.646	24.344	1.730

3	1.596	13.300	45.890	1.181	9.842	34.186	1.296

4	1.244	10.369	56.259	1.029	8.574	42.760	1.137


*Note*. Extraction method: Maximum Likelihood; Rotation: oblimin with Kaiser normalization; N = 200.

**Table 4:** Pattern and Structure Matrix for the Exploratory Factor Analysis.


TASK SCORE	PATTERN MATRIX				STRUCTURE MATRIX			
	
	FACTOR				FACTOR			
	
	1	2	3	4	1	2	3	4

SH-switching cost			0.362				0.375	

SH-mixing cost congruent			0.749				0.748	

SH-mixing cost incongruent			0.686				0.664	

RAT-accuracy								

RAT-RT		0.305				0.327		

AF-sum of switches								

AF-switch rate								

AF-switch rate RT	0.993				0.991			

MATH-flexibility accuracy				0.755				0.772

MATH-control accuracy				0.613				0.607

MATH-flexibility RT		0.857				0.850		

MATH-control RT		0.899				0.893		


*Note*. AF = Ambiguous Figures Task; RAT = Remote Associates Test; SH = Set-Shifting Task; MATH = Math Task; RT = response time; Results obtained using the Maximum Likelihood extraction method with oblimin rotation; N = 200.

In both the pattern matrix and the structure matrix (see Table 4), Factor 1 exhibits strong positive loading for AF switch rate RT (loading = .993). Factor 2 exhibits strong positive loadings from two variables: Math flexibility RT (loading = .857) and Math control RT (loading = .899), and a weaker loading from RAT RT (loading = .305).

Factor 3 exhibits positive loadings from three variables from the SH task: weaker for the switching cost (loading = .362), and stronger for the mixing cost congruent (loading = .749), and the mixing cost incongruent (loading = .686).

Factor 4 exhibits a strong positive load from the Math flexibility accuracy (loading = .755) and Math control accuracy (loading = .613).

We have also conducted two separate EFA analyses by country, with the same specifications as the one on the entire sample. While there are, in some places, inversions compared with the general one and a few differences on loadings, there are not major differences that would suggest essential differences by country. As such we report these analyses broken down by country and their interpretations in Supplementary File 2 – https://figshare.com/articles/figure/Supplem_2/27178947?file=49638681.

Regarding *the relationship between Math Anxiety and the Math Task results*, there is one significant relation. Because this analysis does not pertain directly to the main question of our study, we present its details in the Supplementary File 3 – https://figshare.com/articles/figure/Supplementary_File_3/26206922.

## Discussion

The main objective of the present exploratory study was to investigate whether cognitive flexibility is a domain-general mechanism or a domain-specific property. In other words, we wanted to understand if cognitive flexibility can be envisaged as an ability, like the executive function set-shifting (Bunge, 2024; Diamond, 2006) or some other kind of a system-wide mechanism, or if it is more likely to be a specific property of cognitive processing within particular areas of psychological functioning (Ionescu, 2012, 2017). To this end, we used four tasks that measure flexibility in four different domains – executive function, language, perception, and mathematics – and analyzed their correlations and the underlying common factors as revealed by an Exploratory Factor Analysis. Additionally, we explored whether mathematics anxiety is linked to lower performance in mathematical flexibility.

*Correlations within tasks* indicate that they were well structured and that subjects gave few erratic responses to the items. One exception is the RAT where the lack of a correlation between accuracy and RT might be expected because it is considered an insight problem solving task which depends on many factors, including the item language, language difficulty, and item difficulty (Behrens & Olteţeanu, 2020). In this case, response times and accuracy vary considerably between participants. As such, these results point to measures that can be used for assessing performance in the chosen domains.

Regarding *correlations between tasks*, these do not support the idea of an overall common mechanism. As presented above, correlations did not entail the majority of variables altogether and were small. Moreover, the effect sizes for the significant correlations being very small, we cannot assume very strong relations among the variables.

The switching cost in the SH Task, which is the main measure for set-shifting and expresses task set reconfiguration (Smith et al., 2019), did not correlate with measures in the other tasks. This might be surprising if we take into account that set-shifting is the main proxy for cognitive flexibility in the EF literature (Diamond, 2006, 2013). However, if we consider alternative views, it becomes easier to comprehend the present findings. Specifically, if flexibility is viewed as a property of subsystems in the cognitive system (Ionescu, 2012, 2017) then measures that tap different subsystems would not correlate highly.

A particularly interesting result is the lack of correlation between the variables in the SH Task and the AF Task, a surprising result if we think about these tasks as potentially being part of the same domain, i.e. the perceptual one. However, this is in line with the literature on ambiguous figures. Set-shifting has not been found to relate to switching rate across a variety of ambiguous figures in studies with adults (the Necker cube; Díaz-Santos et al., 2017), ambiguous structure-from-motion (Chamberlain et al., 2018) or in studies with children (with the Duck/Rabbit or ambiguous structure-from-motion; Taranu et al., 2019).

The correlations between accuracy in the RAT and in the Math Task might hint to a link with general intelligence and diligence, especially because the RAT performance only correlates with control items accuracy in Math, reflecting, perhaps, task perseverance (Galla et al., 2014). The link between these two tasks does not seem to point to a general capacity for flexible cognition, as would be the case if RAT performance correlated with performance on the flexible items from the Math Task. These two tasks also had significant correlations in their response times. In other words, subjects who were fast in the RAT Task were also fast in the Math Task, suggesting individual differences in speed of processing. As such, this group of correlations hint to basic cognitive processing commonalities, but these do not implicate a general-purpose process for flexibility. Instead, they suggest that people who tend to be fast or to persevere at one task also tend to be fast or persevere at another.

RAT accuracy also correlated significantly with the reaction time for switching rate in the AF task. A relationship between switching interpretations in the ambiguous figure images and insight problems has been found previously and it has been proposed that they share similar cognitive processes (Schooler & Melcher, 1995) such as conflict monitoring (Wiseman et al., 2011). In early research with ambiguous figures it was even proposed, for example, that the duck-rabbit illusion could be a measure of creativity (Jastrow, 1900) and recent work has found that being exposed to ambiguous figures increases insightfulness (Laukkonen & Tangen, 2017). The relationship is proposed to depend on the conflict monitoring system. Specifically, the conflict experience of seeing two competing interpretations in an ambiguous figure is similar to the conflict we experience in a verbal insight problem. In order to switch perspectives in both tasks and overcome the conflict we may engage the same conflict monitoring system (Laukkonen & Tangen, 2017).

The last significant correlation, between the reaction time for switching rate in the AF task and the flexibility accuracy in the Math Task, may point to the fact that participants whose perceptual switching is slower (higher RTs) were at the same time more accurate in the Math task with flexible items hinting to the need of time for flexibility with perceptions.

Overall, these correlations do not suggest that set-shifting serves as the primary mechanism reflecting cognitive flexibility in diverse domains. They also do not point to an alternative, single mechanism (i.e., as would have been the case if the RAT, AF Task and Math Task performance all correlated). As such, cognitive flexibility might reflect domain-specific properties of processing in those particular domains, with multiple mechanisms in interaction (Ionescu, 2012). While a correlational analysis cannot identify the mechanisms involved, the lack of strong correlations nevertheless points to potentially task-specific flexible processing. These results support two perspectives on cognitive flexibility. The first perspective is that cognitive flexibility is a property (Ionescu, 2012, 2017) and our results seem to indicate that flexibility is not a system-wide property but rather a domain-specific one (i.e., a property of cognitive processing in a specific domain or a property of a specific cognitive process like language or math, Ionescu 2017). The second perspective is the one from research on cognitive control which shows that flexible cognitive control, i.e., the interaction of multiple brain mechanisms during solving a task, leads to adaptive behavior (O’Reilly, 2006; Rougier et al., 2005). In both perspectives, there is no single mechanism or ability that represents flexibility, but multiple mechanisms in interaction that lead to the emergence of flexibility.

An interesting potential third alternative is what Endress (2019) calls “domain-bound mechanisms”. He argues that it is possible that the same core mechanism was duplicated during evolution and has independent copies in different domains which leads to low correlations among tasks. Therefore, these core mechanisms become specialized mechanisms that are neither domain-specific nor domain-general anymore. Further studies are needed to investigate which one of the above-mentioned possibilities better explains the nature of cognitive flexibility.

The Exploratory Factor Analysis also shows that there is no common factor on which all four tasks load. The four factors show high loadings from variables of a single task and, in the case of Factor 2, a small loading from just one other variable from a different task. As such, the factors can be thought of as relatively task-specific, with the existent commonality pointing to basic cognitive processing not specific to flexibility per se. In particular, Factor 2 might be envisaged as involving speed of processing for the domains of language and mathematics, reflecting the fact that subjects who are fast in one domain, such as insight problems, tend to be fast in math problem solving as well.

Generally, the flexibility needed for each task is not shared across tasks. Specifically, there is the flexibility of switching and multitasking for the Set-Shifting Task; the flexibility of finding shortcuts in the Math Task (for flexible items); and the flexibility of finding several interpretations for the same perceptual pattern in the AF Task. The high-level similarity across the tasks, i.e. the need to find and use alternatives in each investigated domain, leads one to wonder if they involve the same “cognitive flexibility” or some kind of general flexibility. In an interesting reflection on the development of executive functions, Morra and colleagues (2018) suggest that cognitive flexibility is a broader concept than set-shifting and point to the difficulty of finding a general ability of flexibility. While some studies have found evidence for separate types of flexibilities like reactive flexibility and spontaneous flexibility (Arán Filippetti & Krumm, 2020), others have not (Baudier, Gros, & Clement, 2023). We suggest that considering flexibility as a domain-specific characteristic of processing is a more appropriate way to conceptualize flexibility (Ionescu, 2012, 2017). This is also in line with the idea of considering flexibility as a consequence of cognitive control and not as a mechanism of it.

### Limits and future research

One potential limitation of this study is the fact that it was delivered as an online experiment. Although it has been a convenient and cost-effective means for us to conduct the experiment in this way, one significant drawback is the potential lack of control over participants’ environments. Variables such as ambient noise, interruptions, and varying screen sizes can introduce unwanted noise into the data, potentially influencing the reliability and validity of the results. Additionally, online experiments may face challenges in ensuring participants’ compliance with task instructions, as there is limited direct supervision. Although the correlations within variables of the same tasks indicates that the participants’ behaviors were not erratic, it is still possible that participants might have behaved differently in a laboratory setting.

It is important to also acknowledge that the study used only one specific task to assess set-shifting abilities. There is a diversity of set-shifting tasks with varying cognitive demands and nuances. As a result, alternative measures may yield different outcomes.

Our sample may have also influenced the results. The small number of male subjects and the fact that they were first year undergraduate students, predominantly psychology majors, might affect how they responded to the tasks.

Lastly, while the small effect sizes both for MANOVA and for the correlations may suggest that there is support in this exploratory study for the idea of not having a single mechanism that represents flexibility, caution is needed because there might be subtle differences between the two country subsamples that need further investigation.

Future endeavors may use several measures for different executive functions, including working memory, inhibition, and conflict monitoring, or cognitive batteries that capture executive processing while exerting cognitive control. Inhibition in particular seems to be one basic executive mechanism that might be identified as a common mechanism across different domains. While this does not directly address the question of whether cognitive flexibility is the same as set-shifting, it would nevertheless pinpoint other basic mechanisms that are needed for flexibility to emerge.

Another approach would be to study flexibility with different experimental tasks in relationship to openness to experience. This would link cognitive flexibility to personal traits. One could also test general anxiety to see how this relates to flexibility in different domains and whether there are some general explanations if the connection is there (Byron & Khazanchi, 2011).

Lastly, replications are needed both for this age group, and also in other populations like adolescents and adults with a larger age range. Future replications are important because our first exploratory step here outlines the fact that cognitive flexibility might not be the same with shifting. If this proves to be the case, researchers need to investigate flexibility in multiple domains and to pinpoint what leads to flexible responses in those specific areas. Because flexibility is considered essential for creative and innovative behavior, a fuller understanding of flexibility will allow us to foster creativity in a society that is increasingly calling for it in everyday life.

A final essential issue that needs to be addressed directly in future studies is whether “cognitive flexibility”, “psychological flexibility”, and simply “flexibility” express the same underlying construct. This seems naturally to be the case, but once again when one looks at the literature they are sometimes used interchangeably and sometimes not. More importantly, if flexibility proves to be a domain-specific property then the appropriate expressions to use will be “attention flexibility”, “rule-use flexibility”, “representational flexibility”, “language flexibility”, “mathematical flexibility” and so on. In each of these cases attaching the word “flexibility” will be justified by situations in which the mentioned process or mechanism is flexible in its deployment.

## Conclusion

The present study adds to a literature that is troubled by terminological confusions and experimental incongruencies with regard to cognitive flexibility. Specifically, the current results suggest that flexibility has strong task-specific and domain-dependent components, rather than reflecting a single cognitive mechanism that robustly appears across multiple tasks that all involve flexibility. More studies are needed to clarify the construct of cognitive flexibility, especially now that the conceptualizations of cognitive flexibility seem to multiply instead of converging toward consensus (Ionescu, 2022; Morra et al., 2018).

## Appendix

Complete list of stimuli for the RAT, the Ambiguous Figures Task, and the Math Flexibility Task (in the Shifting Task there were only red circles and green triangles).

**1. Stimuli for the Remote Associates Test** 

**Table:**

ENGLISH STIMULI				

WORD 1	WORD 2	WORD 3	CORRECT ANSWER	TASK PHASE

rocking	wheel	high	chair	training

aid	rubber	wagon	band	test

flake	mobile	cone	snow	test

cracker	fly	fighter	fire	test

safety	cushion	point	pin	test

fish	mine	rush	gold	test

political	surprise	line	party	test

measure	worm	video	tape	test

sense	courtesy	place	common	test

worm	shelf	end	book	test

flower	friend	scout	girl	test

hound	pressure	shot	blood	test

fly	clip	wall	paper	test

tank	hill	secret	top	test

health	taker	less	care	test

dress	dial	flower	sun	test

**Romanian stimuli** (please refer to Olteţeanu et al., 2019 for the translation of the items in English)				

**WORD 1**	**WORD 2**	**WORD 3**	**CORRECT ANSWER**	**TASK PHASE**

ski	bicicliști	aterizare	pistă	training

replică	ploaie	băutură	acidă	test

vizual	tragere	semantic	câmp	test

ultima	actuală	fixă	oră	test

legitimă	avocat	cheltuieli	apărare	test

ou	pisică	picături	ochi	test

electronică	prioritară	română	poștă	test

iarbă	păr	telefon	fir	test

control	şah	fildeş	turn	test

rotile	electric	birou	scaun	test

electric	cont	literar	curent	test

perete	miez	cocos	nucă	test

muzicală	direcţie	bijuterii	cutie	test

spălat	tuns	cusut	mașină	test

scris	măsurat	muzical	instrument	test

ac	centură	naţională	siguranţă	test

**2. Stimuli for the Ambiguous Figures Task** 

**3. Stimuli for the Math Flexibility Task** 

**Table:**

EQUATIONS	CORRECT ANSWER	TYPE OF ITEM	TASK PHASE

9 + 8 + 7 + 11 + 12 + 13	60	flexible	training

9 + 7 + 6 + 10 + 11 + 13	56	control	training

8 + 2 + 3 + 8 + 2 + 4 + 8 + 2 + 3	40	flexible	test

2 × 3 + 2 × 3 + 2 × 3 + 2 × 3	24	flexible	test

13 × 3 – 2 × 13	13	flexible	test

17 + 22 – 8 – 9	22	flexible	test

7 × 6 × 3 × 0 + 4 × 5 × 4	80	flexible	test

4 × 21 – 3 × 7 × 2	42	flexible	test

6 + 7 + 65	78	flexible	test

4 + 5 + 6 + 7 + 6 + 5	33	flexible	test

7 + 2 + 4 + 4 + 2 + 6 + 7 + 3 + 5	40	control	test

1 × 2 + 2 × 2 + 3 × 1 + 4 × 2	17	control	test

13 × 3 – 12 × 2	15	control	test

15 + 18 – 4 – 8	21	control	test

4 × 7 × 2 + 1 × 5 × 3	71	control	test

4 × 19 – 3 × 7 × 2	34	control	test

25 + 17 + 32	74	control	test

9 + 3 + 2 + 8 + 7 + 4	33	control	test
